# A Pilot Study on Baseline Fungi and Moisture Indicator Fungi in Danish Homes

**DOI:** 10.3390/jof7020071

**Published:** 2021-01-20

**Authors:** Birgitte Andersen, Jens C. Frisvad, Robert R. Dunn, Ulf Thrane

**Affiliations:** 1 Division of Energy Efficiency, Indoor Climate and Sustainability of Buildings, Department of the Built Environment, Aalborg University, A.C. Meyers Vænge 15, DK-2450 Copenhagen SV, Denmark; 2 Section for Synthetic Biology, Department of Biotechnology and Biomedicine, Technical University of Denmark, Søltofts Plads 221, DK-2800 Kongens Lyngby, Denmark; jcf@bio.dtu.dk; 3 Department of Applied Ecology, Campus Box 7617, NC State University Campus, Raleigh, NC 27695-7617, USA; rrdunn@ncsu.edu; 4 Wood and Biomaterials, Building and Construction, Danish Technological Institute, Gregersensvej 1, DK-2630 Taastrup, Denmark; ult@teknologisk.dk

**Keywords:** *Aspergillus*, *Cladosporium*, *Penicillium*, ITS1 sequencing, DG18, V8, baseline spora, building mycobiota, indoor fungi

## Abstract

In many complaint cases regarding bad indoor environments, there is no evidence of visible fungal growth. To determine if the problems are fungi-related, dust sampling is the method of choice among building surveyors. However, there is a need to differentiate between species belonging to a normal, dry indoor environment and species belonging to a damp building envelope. The purposes of this pilot study were to examine which fungal species are present in problem-free Danish homes and to evaluate different detection and identification methods. Analyses showed that the fungal diversity outside was different from the diversity inside and that the composition of fungal species growing indoors was different compared to those found as spores, both indoors and outdoors. Common for most homes were *Pseudopithomyces* *chartarum*, *Cladosporium* *allicinum* and *Alternaria* sect. *Infectoriae* together with *Botrytis* spp., *Penicillium* *digitatum* and *Pen*. *glabrum*. The results show that ITS sequencing of dust samples is adequate if supported by thorough building inspections and that food products play as large a role in the composition of the baseline spora as the outdoor air and surrounding vegetation. This pilot study provides a list of baseline fungal species found in Danish homes with a good indoor environment.

## 1. Introduction

Fungal spores are ubiquitous, but not all fungal species can grow everywhere. In water-damaged buildings, *Penicillium chrysogenum* is almost omnipresent because it can grow on both wet and semi-dry materials [1], whereas *Stachybotrys chartarum* is restricted to very wet gypsum wallboard, wallpaper and cardboard [2]. Much is known about the mycobiota or fungal biota or fungal “flora” or funga (fungal species in active growth [1]) in damp or water-damaged buildings [3,4,5,6], but less is known about the spora or fungal diversity (fungal species present as dormant spores [7]) in the air and on surface dust in buildings that have no humidity problems and no dissatisfied occupants [8,9].

Sampling, detection and identification methods to determine the fungal spora in indoor environments at the species level have always been problematic and the subject for discussion and controversy: e.g., air sampling versus material sampling, culture methods versus molecular methods or morphology versus gene sequencing. Older studies using culture methods on air and dust samples reported fungal identity at the genus or species group level (e.g., *Penicillium* or *Aspergillus/Penicillium* group) [10,11,12]. Newer studies using next-generation sequencing on dust and swab samples reported fungal identity at the genus level [13], but mostly at the order or class level (e.g., *Eurotiales* or *Eurotiomycetes*) [14,15,16], or even at the phylum level [17].

Further complications in fungal identification have arisen in the last decade. Many name changes have occurred with the increased use of molecular tools and the adoption of the International Code of Nomenclature for algae, fungi and plants in 2017 [18]. Many genera have changed names, some genera have merged, and others have been split into several new ones [6]. However, current and correct fungal identification to the species level is necessary to distinguish between mycobiota and spora, to explore their origin, and to compare their properties in the scientific literature. 

In a water-damaged home with visible fungal growth, action is usually straight forward (i.e., repair/renovation), but often occupants complain about their indoor environment without there being any visible signs of fungal growth. Fungal growth indoors can, however, be invisible to the naked eye: e.g., *Chaetomium globosum* on the reverse of a wet gypsum wall or *Aspergillus versicolor* under the floorboards. But an unsatisfactory indoor environment can also have other causes than fungal growth: e.g., off-gassing of chemicals from furniture and building materials. In either case, dust sampling becomes the proxy method of choice for building surveyors to determine the cause of the complaint. When the identity of the fungal species in a dust sample is known, the source of origin can often be inferred. Studies show that geographic location, climate zone and outdoor air [13,19] are determining factors for the spora in indoor air and dust. But what is the normal fungal spore diversity or baseline spora in indoor dust in Danish homes?

Denmark is an agricultural country located in the temperate oceanic climate zone with distinct seasons. Outside, there are 15–30 times as many viable fungal spores in the air in summer compared to the wintertime [20]. The indoor spora might reflect these outdoor patterns in the summer, but in wintertime, during which Danes rarely air their homes [21], the outdoor and indoor spora are expected to diverge. Therefore, this small pilot study was conducted in the winter to determine if it is possible to find what constitutes a normal Danish fungal spore diversity (the baseline spora) in indoor air and subsequently in dust and to pinpoint some of the factors that shape them. One hypothesis is that fungi brought into homes, e.g., on the food products we buy, play a role in the overall picture of the Danish spora indoors. The aims of the study were (1) to determine the fungal spore diversity in dust in and between private homes that were perceived to be dry and healthy by their owners, (2) to infer the origin of the dominant fungal genera/species found, and (3) to compare detection (culture-dependent and -independent) and identification (morphology and ITS sequencing) methods.

## 2. Materials and Methods

The study was inspired by a previous study by Dunn et al. [22]. Samples were taken both indoors and outside nine private homes that were perceived as dry, without visible fungal growth and with no indoor environment problems according to the owners. The samples were analyzed using both culture-dependent methods and culture-independent methods, because both methods are used by building inspectors in Denmark during a house inspection. Each home was also subjected to an inspection for visible fungal growth at the same time as the samples were taken and later revisited if data treatment showed unusual findings.

### 2.1. Sampling Locations

Samples were taken from the nine private homes between 21 January 2015 and 18 March 2015. In this period, the weather was fairly dry (ca. 56 mm in total) and mild (min./max. −9/14 °C, average ca. 2.4 °C) compared to the climate norm for 2006–2015 and with no snow cover. The nine homes were located in North Zealand and the Greater Copenhagen area (Table 1) with ca. 50 km between the most northerly and southerly homes in rural as well as suburban and urban settings. None of the homes had mechanical ventilation systems.

### 2.2. Sampling and Treatment Protocols

Samples for both DNA and culture were taken on the upper doorframe on the outside surface of the front door and on the upper doorframe on an interior door in the hallway of the residence. These sampling sites were selected because they are not likely to be cleaned frequently and therefore can serve as passive reservoirs for environmental dust and particles. Swab samples were taken for both DNA sequencing (dual-tipped sterile polyester swab (BD, Le Pont-de-Claix, France)) and culturing (single-tipped sterile cotton swab (Deltalab, Barcelona, Spain)) both outside and in accordance with the following protocol: the sampling area was first swabbed with the DNA swab (one wipe with both tips touching an area of ca. 1 cm × 10 cm), followed by the culture swab of approximately the same area (four wipes rolling the tip forwards and backwards on area of ca. 1.5 cm × 10 cm). Immediately after being returned to the lab, the culture swabs were streaked out onto Petri dishes and the DNA swabs were stored in the freezer until shipment to the Cooperative Institute for Research in Environmental Sciences, University of Colorado at Boulder, Boulder, CO, USA.

### 2.3. Additional Samples

None of homes had—according to the owner—any humidity problems, but three had a history of water damage (Table 1). The damage had been repaired well before samples were taken. However, each home was examined for any visible mold growth, which was found in two cases (Table 1), and additional swab samples were taken from the moldy areas and treated according to the protocol mentioned earlier. During the course of the sampling, a student flat in Kgs. Lyngby experienced water damage resulting in massive mold growth in the bathroom, which was constructed of gypsum wallboard. Two moldy areas were sampled and treated according to protocol. To match these samples, two samples of new gypsum wallboard with mold growth from an ongoing experiment by Andersen et al. [2] were also included and treated according to protocol. 

### 2.4. Culture-Independent Method: DNA Sequencing

DNA swabs (25 in total) were sent to Cooperative Institute for Research in Environmental Sciences, for extraction, PCR, and sequencing. At the Cooperative Institute, swab tips were placed into 2 mL 96-well plates (Axygen Inc., Union City, CA, USA). Plates were processed using the Extract-*N*-Amp PCR kit (Sigma-Aldrich, Inc., St. Louis, MO, USA), following a modified version of the manufacturers’ instructions (as in Barberan et al. [23]). The first internal transcribed spacer (ITS1) region of the rRNA operon was sequenced, using the ITS1-F (CTTGGTCATTTAGAGGAAGTAA) and ITS2 (GCTGCGTTCTTCATCGATGC) primer pair. The primers included the appropriate Illumina adapters, with the reverse primers also having an error-correcting 12 bp barcode unique to each sample to permit multiplexing of samples. PCR products from all samples were quantified using the PicoGreen dsDNA assay and pooled together in equimolar concentrations for sequencing on an Illumina MiSeq instrument. 

### 2.5. Data Treatment

A total of 1312 operational taxonomic units (OTUs), each trimmed to 251 base pairs, were returned from the Cooperative Institute for Research in Environmental Sciences with identities from the UNITE database. All sequences were first clustered using Clustal Omega (EMBL-EBI, http://www.ebi.ac.uk, standard settings) to facilitate initial work on excluding non-fungal and unidentified fungal sequences. Clusters of OTUs identified as virus and bacteria in GenBank and outliers of fungal OTUs with no identification were excluded, as were fungal OTUs where there was no agreement between UNITE and GenBank (206 OTUs in total). The identity of each OTU according to the UNITE database was manually cross-checked with GenBank (NCBI) using the “Percent identity” and “Distance Tree of Results” functions after three BLAST runs: (1) including “All sequences”, (2) excluding “Uncultured/environmental sample sequences”, and (3) limited to “Sequences from type of material” and only comparing the identity with sequences from cultures with strain numbers from well-known curated fungal collections (Supplementary Table S1) and type cultures, if possible. Synonyms and current fungal names were checked and adopted from Index Fungorum (http://www.indexfungorum.org), Mycobank (http://www.mycobank.org/) and the current taxonomic literature for the most frequently occurring genera and species and for all food and indoor fungi in the dataset. OTUs were pooled if UNITE, GenBank, FunCBS (Westerdijk Institute, Utrecht, The Netherlands) and Indoor.txt (Westerdijk Institute, Utrecht, The Netherlands) agreed on species identity and the sequences were located in the same cluster according to the Clustal Omega dendrogram. When two or more fungal species names of strains from curated collections were equally likely, identity was set at the section or clade or complex level. Selected OTU sequences were rechecked in November 2020 (Supplementary Table S1). Unscrambler (version X 10.4, CAMO) was used to perform principal component analysis (PCA) on a reduced and pooled dataset (18 indoor and outdoor samples × 41 common genera, species complexes, sections, series, or species indoors and out (29 marked with * in Table 2 and Table 3)). The PCAs were done with standardization (X/SDev) and with log-transformation (X = log(X + 1)) followed by standardization (X/SDev).

**Table 2:** Culture-independent identification. The 15 most abundant fungal genera outdoors and indoors at the nine homes (H) and the total reads for the different pooled operational taxonomic units (OTUs). High occurrence indoors compared to outdoors and vice versa are in bold.

Genus	Total DNA Reads		Outdoors									Indoors								
	Out	In	H-1	H-2	H-3	H-4	H-5	H-6	H-7	H-8	H-9	H-1	H-2	H-3	H-4	H-5	H-6	H-7	H-8	H-9
*Alternaria*	2785	**7596**	0	908	189	225	50	452	248	7	706	244	461	865	1931	55	0	2418	1619	3
*Aspergillus*	364	**6784**	1	6	36	20	1	269	17	0	14	0	2767	405	386	2772	101	295	57	1
Aureobasidium *	**3724**	1893	149	1259	4	990	22	5	1079	0	216	24	239	245	509	110	0	555	211	0
Blumeria *	**4275**	1837	147	559	590	146	32	1569	324	5	903	535	5	244	0	0	0	0	1053	0
Botrytis *	1488	**5652**	0	711	0	1	4	701	71	0	0	594	3	702	831	0	1832	942	748	0
*Cladosporium*	5193	**10,645**	67	1202	327	1558	52	579	614	56	738	1110	1942	1297	2023	173	57	1313	2666	64
Devriesia *	**3740**	410	130	965	124	301	1896	79	220	0	25	0	89	65	41	78	0	89	48	0
Exophiala *	**7198**	129	0	124	510	773	224	4395	438	17	717	0	0	39	47	0	0	0	43	0
Itersonilia *	**3242**	1693	7	11	1440	534	48	25	1168	8	1	439	7	822	4	0	0	214	207	0
Knufia *	**2618**	234	0	0	247	320	84	38	867	0	1062	0	86	50	0	0	0	0	98	0
*Penicillium*	466	**5103**	0	52	42	1	0	139	162	9	61	1065	258	480	179	2081	0	908	43	89
Pseudopithomyces *	264	**7069**	69	82	0	7	9	0	97	0	0	97	545	1988	1626	0	0	1728	1085	0
Scoliciosporum *	**5317**	25	108	139	911	160	4	0	85	1	3909	22	0	1	0	0	0	0	2	0
Taphrina *	**3420**	353	0	0	50	282	1235	53	71	0	1729	29	0	165	0	0	0	95	64	0
Wallemia *	1	**746**	1	0	0	0	0	0	0	0	0	0	107	0	0	548	0	91	0	0
**Total DNA reads ^†^**	104,858	85,701	5853	15,950	10,571	10,491	17,566	14,328	13,337	761	16,001	8928	11,366	17,046	10,082	8604	5977	12,245	11,273	180

**Figure 1:**
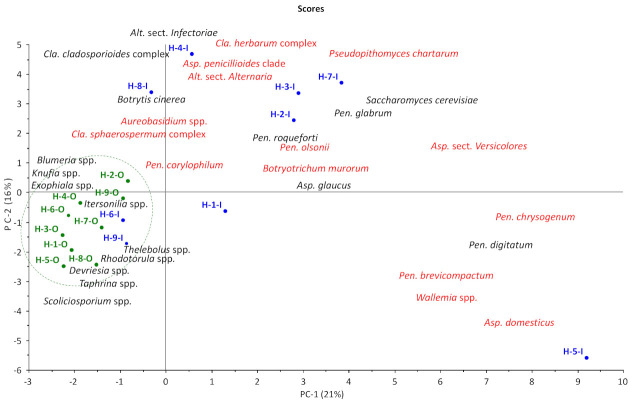
Score plot from a PCA (18 × 41) based on the 9 homes (H) (indoors (I) in blue and outdoors (O) in green) and 41 of the most common fungal genera and species. Selected fungal genera and species have been transferred from the biplot. Fungal species in red are humidity indicator fungi. The plot axes PC-1 and PC-2 are principal components with arbitrary score values. *Alt.: Alternaria; Asp.: Aspergillus; Pen.: Penicillium. Cladosporium* species as complexes (*Cla*. *herbarum* complex: *Cla*. *allicinum* and *Cla. limoniforme*; *Cla*. *sphaerospermum* complex: *Cla*. *dominicanum*, *Cla*. *halotolerans* and *Cla*. *sphaerospermum*).

* Included in the PCA analysis in Figure 1. ^†^ Total reads for all OTUs outdoors, indoors and for each sample site.

**Table 3:** Culture-independent identification. The most abundant indoor fungal species at the nine homes (H) and the total reads for the different OTUs out and in. Samples from the interior doorframe and on three samples from painted brick wall with visible fungal growth (G). High occurrences on the building materials compared to the corresponding dust sample are in bold.

Species	Total DNA Reads		Indoors									Fungal Growth		
	Out	In	H-1	H-2	H-3	H-4	H-5	H-6	H-7	H-8	H-9	H-3-G	H-4-Ga	H-4-Gb
*Acremonium charticola*	5	59	0	0	0	**0**	0	59	0	0	0	0	**3483**	50
*Akanthomyces lecanii*	0	0	0	0	0	**0**	0	0	0	0	0	0	0	**1289**
*Alternaria* sect. Alternaria *	1542	3275	119	161	494	258	1	0	1547	694	1	0	2	0
*Alternaria* sect. Infectoriae *	1201	4051	125	297	360	1656	0	0	826	786	1	0	0	0
*Alternaria* sect. *Ulocladium*	14	59	0	3	0	0	55	0	0	0	1	0	0	0
*Aspergillus canadensis*	64	361	0	271	0	**0**	0	0	89	0	1	1	**1856**	0
Aspergillus domesticus *	221	2829	0	79	27	0	2550	40	100	33	0	0	0	0
*Aspergillus flavus*	15	17	0	0	0	17	0	0	0	0	0	0	0	0
*Aspergillus fumigatus*	40	37	0	0	37	0	0	0	0	0	0	0	0	0
Aspergillus glaucus *	1	2111	0	1980	0	0	83	1	47	0	0	0	0	0
*Aspergillus hiratsukae*	1	2	0	0	1	0	0	0	0	1	0	1	0	0
*Aspergillus niger*	0	15	0	0	14	0	0	0	0	1	0	0	0	0
*Aspergillus penicillioides* clade *	0	91	0	0	71	0	0	0	20	0	0	0	0	0
*Aspergillus salinarum*	11	80	0	80	0	0	0	0	0	0	0	0	0	0
*Aspergillus* series Versicolores *	0	428	0	127	119	**0**	139	0	38	5	0	0	**1620**	**4108**
*Aspergillus vitricola*	4	813	0	230	136	369	0	60	1	17	0	0	0	1
*Aspergillus westerdijkiae*	7	0	0	0	0	0	0	0	0	0	0	0	0	0
Botryotrichum murorum *	11	269	0	233	0	0	0	0	36	0	0	0	0	0
*Chaetomium globosum*	19	69	0	60	9	0	0	0	0	0	0	0	0	0
Cladosporium allicinum *	2700	6909	588	1636	655	982	115	56	893	1984	0	110	0	0
*Cladosporium cladosporioides* complex *	1110	3222	522	182	589	835	58	1	353	618	64	0	0	0
Cladosporium dominicanum *	264	140	0	100	1	**38**	0	0	0	1	0	0	**476**	2
Cladosporium halotolerans *	805	72	0	24	**0**	0	0	0	48	0	0	**33**	14	0
Cladosporium sphaerospermum *	29	185	0	0	38	**139**	0	0	0	8	0	0	**8172**	5
*Debaryomyces hansenii*	203	704	125	116	**27**	**0**	395	0	27	14	0	94	**1386**	**1536**
*Monocillium tenue*	12	32	0	0	0	**0**	0	0	32	0	0	0	0	**8540**
*Penicillium adametzioides*	0	112	0	0	0	112	0	0	0	0	0	0	0	0
*Penicillium bialowiezense*	10	21	0	0	7	0	14	0	0	0	0	0	0	1
Penicillium brevicompactum *	103	1116	233	0	48	0	744	0	81	10	0	0	0	7
Penicillium chrysogenum *	44	909	16	230	67	45	409	0	138	4	0	0	1	1
*Penicillium citreonigrum*	23	138	0	0	0	13	0	0	36	0	89	0	0	0
*Penicillium citrinum*	0	69	0	0	0	0	69	0	0	0	0	0	0	0
Penicillium corylophilum *	149	0	0	0	0	**0**	0	0	0	0	0	0	0	**115**
Penicillium digitatum *	48	2114	763	28	190	2	750	0	377	4	0	0	0	0
Penicillium glabrum *	23	511	53	0	113	7	84	0	235	19	0	0	0	0
*Penicillium lanosum*	0	10	0	0	0	0	10	0	0	0	0	0	0	0
Penicillium olsonii *	0	62	0	0	55	0	1	0	0	6	0	0	0	0
*Penicillium oxalicum*	7	0	0	0	0	0	0	0	0	0	0	0	0	0
Penicillium roqueforti *	7	41	0	0	0	0	0	0	41	0	0	0	0	0
*Penicillium roseopurpureum*	0	0	0	0	0	**0**	0	0	0	0	0	0	0	**2156**
Saccharomyces cerevisiae *	11	1266	0	0	415	146	252	0	423	30	0	0	0	0
*Stachybotrys chartarum*	48	38	0	0	0	0	0	0	0	38	0	0	0	1
*Verrucocladosporium dirinae*	243	97	0	0	26	**0**	71	0	0	0	0	0	**352**	0
*Wallemia ichthyophaga*	0	24	0	0	0	0	0	0	24	0	0	0	0	0
*Wallemia muriae*	1	722	0	107	0	0	548	0	67	0	0	0	0	0

* Included in the PCA analysis in Figure 1. *Cladosporium* species as complexes (*Cla*. *herbarum* complex: *Cla*. *allicinum*; *Cla*. *sphaerospermum* complex: *Cla*. *dominicanum*, *Cla*. *halotolerans* and *Cla*. *sphaerospermum*).

### 2.6. Culture-Dependent Method: Fungal Growth, Identification and Enumeration

Each growth swab was streaked out onto two Petri dishes with V8 (Campbell’s V8 juice agar [6] with chloramphenicol (0.05 g/L) and chlortetracycline (0.05 g/L)) and two Petri dishes with DG18 (Dichloran 18% Glycerol agar [6]). The Petri dishes were incubated at 20 °C and read after 7 and 14 days. After 7 days of growth on DG18 and V8 media, the plates were photographed and fungal and yeast colonies counted. Colonies were identified to genus level using a dissection microscope and representative fungal colonies were isolated and inoculated for species identification. Fungal colonies were identified to species level using a compound microscope: *Alternaria* were identified according to Simmons [24], *Aspergillus* and *Penicillium* according to Samson et al. [6], Valdez et al. [25], Chen et al. [26], *Chaetomium* according to Wang et al. [27], *Cladosporium* according to Bensch et al. [28] and other fungi according to Samson et al. [29]. Pictures were taken of each Petri dish and non-sporulating colonies were marked and checked again for sporulation after 14 days of growth. After species identification, each colony forming unit (CFU) in the pictures were annotated with species ID and counted across all samples. Non-sporulating colonies were transferred to malt extract agar (MEA, [6]) and labelled “Mycelia sterilia” if no spores were formed after 14 days.

## 3. Results

### 3.1. Culture-Independent Methods (DNA Sequencing)

#### 3.1.1. OTU Diversity and Abundance

A total of 1106 fungal operational taxonomic units (OTUs) were found across all 21 samples (nine indoor samples, nine outdoor samples and three samples with fungal growth). The fungal diversity and abundance were greater outdoors than indoors. Across the nine outdoor samples, 732 fungal OTUs (104,858 reads) were found, compared to 591 fungal OTUs (85,701 reads) for the nine indoor samples. Outdoors, the diversity and abundance of ascomycetes (468 OTUs/73,873 reads) were both greater than those of basidiomycetes (208 OTUs/18,793 reads), lichens (46 OTUs/11,507 reads), and mucoromycetes (10 OTUs/685 reads). Indoors, the diversity in ascomycetes and basidiomycetes was almost the same (284 and 283 OTUs, respectively) despite the greater number of reads of ascomycetes than of basidiomycetes (60,722 and 24,130 reads, respectively). Lichens (16 OTUs/327 reads) and mucoromycetes (8 OTUs/168 reads) were neither diverse nor particularly abundant. Samples from areas with fungal growth were quite different from both outdoor and indoor samples. Across the three samples of a painted brick wall with fungal growth, ascomycetes were represented by many reads, but a low diversity of OTUs (35 OTUs/35,568 reads) was observed, and the occurrence of basidiomycetes was negligible. Across the four gypsum wallboard samples with fungal growth, the diversity and read numbers of ascomycetes was higher than for the growth on brick (66 OTUs/67,522 reads), but lower than for dust samples. Generally, dust samples were characterized by high diversity (hundreds of OTUs), whereas areas of fungal growth were characterized by lower diversity (tens of OTUs). 

#### 3.1.2. Genus Diversity and Abundance

*Exophiala* spp. (7198 reads) was the most abundant genus outside (Table 2), followed by *Scoliciosporum* sp. (5317 reads (as *S. umbrinum*)) and *Cladosporium* spp. (5193 reads). Indoors, *Cladosporium* spp. (10,645 reads) was most abundant, with *Alternaria* spp. (7596 reads) and *Pseudopithomyces* sp. (7069 reads as *Pse. chartarum*) second and third. As can be seen from Table 2, *Exophiala* and *Scoliciosporum* spp. were rarely found indoors (129 and 25 reads, respectively), while *Pse. chartarum* was rare outdoors (264 reads). *Aspergillus, Penicillium* and *Wallemia* spp. were much more abundant indoors (6784, 5103 and 746 reads, respectively) than outdoors (364, 466 and 1 reads, respectively). For the yeasts and yeast-like fungi, *Saccharomyces cerevisiae* and *Cryptococcus* spp. were the most abundant species indoors (1266 and 1019 reads, respectively) compared to outdoors (11 and 425 reads, respectively).

#### 3.1.3. Species Diversity and Abundance Indoors

Some fungal species were found in all homes, whereas other species were located in only one home (Table 3). *Cla. cladosporioides* complex (3222 reads) was the most common *Cladosporium* group and was found in all homes, followed by *Cla. allicinum* (6909 reads) and *Alt.* sect. *Alternaria* (3275 reads), found in eight of the nine homes. *Alt.* sect. *Infectoriae* (4051 reads), *Pen. digitatum* (2114 reads), and *Pen. chrysogenum* (909 reads) were detected in seven of the nine homes. *Pse. chartarum*, the most abundant species indoors, was only found in six out of the nine homes, but with high read numbers when present. Other species, such as *Asp. domesticus* and *Asp. glaucus*, were found in high numbers indoors (2250 and 1980 reads, respectively), but only in one home each (H-5 and H-2, respectively).

Table 3 also shows the results of three samples with visible fungal growth due to local condensation. An inner wall (painted brick wall) in the bathroom at H-3 showed moderate growth with *Cla. allicinum*, *Cla. halotolerans*, and *Debaryomyces hansenii*, while two samples from outer walls (painted brick walls) in a bedroom at H-4 showed massive growth that included *Acremonium charticola*, *Asp. canadensis*, *Asp.* series *Versicolores*, *Cla. sphaerospermum*, *Deb. hansenii*, *Akanthomyces lecanii, Monocillium tenue*, and *Pen. roseopurpureum*. However, none of these fungal species were found in higher amounts in the corresponding dust samples compared to dust samples from the other seven homes (Table 3), except for *Cla. sphaerospermum* in H-4.

The moisture indicator fungus, *Stachybotrys chartarum*, was only found in low numbers and only in one home (38 reads on H-8-I, Table 3). Other moisture indicator fungi, such as *Fusarium, Didymella*, and *Trichoderma* spp., were only detected sporadically indoors and in low numbers (<10 reads) in this study.

Table 4 shows the result of the three brick wall samples with fungal growth from Table 3 compared with four gypsum wallboard samples with fungal growth. The gypsum samples are unrelated to the home study but have been included to enable comparisons between material types. As can be seen, *Sta. chartarum* was found on the gypsum wallboard samples (Ub and Pa), but not on the brick wall samples. Similarly, *Alt.* sect. *Ulocladium, Asp. hiratsukae, Candida parapsilosis*, and *Pen. chrysogenum* were abundant on the gypsum samples, but almost absent on the brick wall samples. On the other hand, *Asp. canadensis*, *D. hansenii*, *Acr. lecanii*, *Mon. tenue*, and *Pen. roseopurpureum* were found on the brick wall samples, but not on the gypsum samples. 

Principal component analysis (PCA) based on the most abundant genera overall and the most abundant indoor species (marked with * in Table 2 and Table 3, *Cladosporium* pooled into complexes) showed that the outdoor samples were more similar to each other than they were to any of the indoor samples (Figure 1). All outdoor sampling sites grouped together due to high reads of genera such as *Blumeria*, *Knufia*, *Devriesia*, and *Taphrina* and low reads of *Aspergillus*, *Penicillium*, and *Pseudopithomyces* (Table 2). Two indoor sampling sites (H-6-I and H-9-I) also grouped with the outdoor sample mostly due to the absence of indoor fungi compared to the other indoor samples. The seven remaining homes were scattered throughout the plot, reflecting apparently idiosyncratic differences from one house to the next. All homes with a history of water damage (H-2, H-7 and H-8) or found to have fungal growth (H-3 and H-4) were located together in the upper part of the plot. H-5-I, located in the bottom right corner, is an outlier due to high reads of *Asp. domesticus*, *Pen. brevicompactum* and *Wal. muriae* compared to other indoor samples (Table 3). 

### 3.2. Culture-Dependent Methods (Growth on DG18 and V8)

#### 3.2.1. Colony Forming Unit (CFU) and Genus Diversity and Abundance

**Figure 2:**
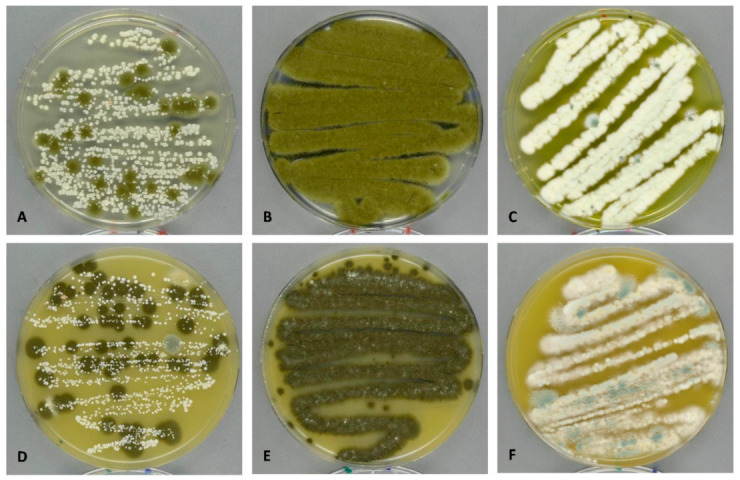
Culture-dependent swab samples taken after DNA swabs on H-3-G (**A**,**D**), H-4-Ga (**B**,**E**) and H-4-Gb (**C**,**F**) with visible growth. (**A**–**C**): DG18 after 7 days at 20 °C and (**D**–**F**): V8 after 7 days at 20 °C.

A total of 467 fungal colony forming units (CFUs) were identified across the 18 samples (nine indoor samples and nine outdoor samples) using DG18 and V8 as culture media. The three swab samples of fungal growth (H-3-G, H-4-Ga and H-4-Gb) were not countable due to high numbers of CFUs and coalescing colonies (Figure 2). The total CFUs for outdoors was not particularly different from the total CFUs found indoors (262 and 205, respectively) and, as expected, both the diversity and the abundance in CFUs were much lower compared to the culture-independent samples. No basidiomycetes or lichens could be identified on DG18 and V8, but there were 45 CFUs with no sporulation (Mycelia sterilia) outside and 25 CFUs inside, which may be basidiomycetes; however, no clamp connections were found either. *Cladosporium* spp. were the most abundant fungi detected outside (87 CFUs in total), while the total CFU for *Cladosporium* spp. inside was much lower (seven CFUs in total) (Table 5). Similar low distributions indoors were seen for *Didymella* and different yeast spp. (zero and two CFUs, respectively). *Penicillium* was the most prevalent genus inside (132 CFUs in total), while the total *Penicillium* CFUs outside was five-fold lower. Similar differences in indoor to outdoor CFU numbers were seen for *Aspergillus* spp. (30 CFUs indoors vs. one CFU outdoors).

#### 3.2.2. Species Diversity and Abundance Indoors

No fungal species was found to be common in all nine homes using the culture methods. *Pseudopithomyces chartarum*, the most prevalent fungus indoors according to the culture-independent method, was not found in any sample using the culture-dependent method. Likewise, *Cla. allicinum* was not found in any culture-dependent sample indoors, while *Cla. cladosporioides* (four CFUs) and *Cla. sphaerospermum* (three CFUs) were found inside three and two homes, respectively, and in very low CFU numbers (Table 5). The most common *Penicillium* species, *Pen. brevicompactum* (16 CFUs) and *Pen. glabrum* (15 CFUs), were found in five of the nine homes, whereas *Pen. digitatum* (69 CFUs) was found in three homes. *Pen. chrysogenum*, which was detected very frequently using the culture-independent method, was only detected in three homes with five CFUs. *Asp. glaucus* and *Asp. domesticus* were not found either, but contrary to the culture-independent method, 20 CFUs of *Asp. niger* were found in home H-3 and 17 CFUs of *Didymella* sp. were found outside home H-8. 

## 4. Discussion

The results of this Danish pilot study show that a closed building envelope shields well against airborne fungal spores from the outside, but at the same time traps spores from fungi that grow or have grown somewhere inside the building. Contrary to studies conducted in Mediterranean or subtropical climates [8,9,13,14], a clear difference between the compositions of fungal species indoors and outdoors was seen. This was also seen in other studies conducted in a Scandinavian or temperate climate [5,30] where households keep windows and doors closed as much as possible during the winter. However, both nature and culture play a role on the fungal composition indoors. In North Zealand, Denmark, where all nine homes are located, there are large expanses with deciduous forests, agricultural fields, and pastures that change with the seasons, resulting in variations in outdoor fungi [20]. Since our results were obtained in the colder winter months, when airing and/or ventilation was sparse, the influx and influence of fungal spores from the outside environment was limited, giving a clearer picture of the fungal species that have indoor sources. In the summertime, the differences would probably be much smaller because it is warm enough to have doors and windows open most of the day. The results show that previous water damages may interfere when culture--independent methods are used, because fungal spores from old damage may linger on for months or perhaps years in undisturbed dust. The results also show that occupants may perceive their home to be problem-free, even though there might be minor undiscovered dampness somewhere in the home, especially in basements or bathrooms.

For some fungal genera, such as *Pseudopithomyces* and *Wallemia*, the source is inside, while for *Blumeria* and *Itersonilia*, an outdoor source is a determining factor. For example, one home (H-1) had slightly higher reads indoors of *Scoliciosporum* spp., which are strictly outdoor lichenized fungi. A second inspection of the home showed that the exterior window ledge outside the bedroom was covered with lichens, showing that the proximity of an outdoor fungal source plays a role in the overall fungal composition indoors. The opposite was the case in another home (H-4) where the bedroom of a teenager had massive growth of, amongst others, *Aspergillus* spp. These fungi were, however, not detected in the dust sample, probably due to the remoteness of the bedroom compared to the interior sampling point. These results show that the proximity of the fungal source, inside or out, plays a role in the overall composition of spores in indoor dust samples.

For genera such as *Aspergillus* and *Penicillium*, which both seem to have indoor sources, species identification is necessary to pinpoint if their source indoors is either food- or building-associated. This was seen in H-1 and H-5, where moldy tangerines were found. High reads and CFUs of *Pen. digitatum* were later detected at both sites. *Pen. digitatum*, which is associated with moldy citrus fruit [6], was detected using the culture-independent method in most homes, but only in three homes using the culture-dependent method. *Pen. glabrum* was also detected frequently indoors due to its association with the brown dried scales found on most onions [6]. *Asp. glaucus* was detected in several homes because of its association with white bread, dried herbs and spices [6], and a very moldy baguette was subsequently found at H-2 after a second inspection. These results show that food-borne fungi play a key role in Danish homes compared to, e.g., American homes [8,13], probably due to differences in food and cooking culture. Many Danes still prepare lunch packs and cook meals from scratch on a daily basis and therefore always have a certain amount of (moldy) bread, fruit and vegetables in their kitchens.

A presence of spores from moisture indicator fungi was seen in the three homes with a history of water damage (H-2, H-7, and H-8). Even though renovation had been completed at least 6 months prior to sampling, low reads of *Botryotrichum murorum*, *Chaetomium globosum*, and *Stachybotrys chartarum*, all indicator fungi for severe water damage [1,27], were still found in one or more homes using the culture-independent method, whereas only one colony of *Cha. globosum* was detected in H-2 using the culture-dependent method. This suggests that culture-independent methods, such as DNA sequencing, can detect fungal spores after old, repaired water damage, due to the longevity of the DNA compared to the viability of the spores, and shows that knowledge of the history of the home is important, in order not to draw the wrong conclusions, i.e., ongoing problems with water damage. On the other hand, at home 5, where no visible fungal growth was discovered and no history of water damage, high reads of *Asp. domesticus*, *Pen. brevicompactum* and *Wallemia muriae* were found with the culture-independent method and some CFUs of *Pen. brevicompactum* using the culture-dependent method. These fungal species are associated with moderate humid indoor environments, especially moist plaster [6], and this fungal combination suggested that this home might have humidity problems, even though the owners considered their home dry and problem-free. A second inspection revealed that a remote part of the basement had elevated moisture levels and a moldy odor, but no visible fungal growth. These findings show that a thorough inspection or even re-examination is important when high reads of several species of moisture indicating fungi are detected.

### 4.1. Detection Methods

Absolute quantitative measurements of fungal diversity and abundance between species are not possible with either culture-dependent (CFUs) or culture-independent (reads) methods. One CFU on a growth medium may be the result of several fresh spores clustering together and thereby giving a false lower count than what is really present. On the other hand, the number of reads may vary between species depending on the number of cells in the spores, giving a false higher count, especially for fungal species such as *Alternaria* and *Epicoccum* [31]. However, comparison between sampling sites (i.e., indoors and out) and relative quantitative evaluation between individual species is possible.

Spore mortality and the ability to grow on the chosen media are probably the two major reasons for the big difference between the results of the culture-independent method (reads) and the culture-dependent method (CFUs). The ITS primer will detect both viable and dead fungal spores, while culture methods select only for living spores. The viability of fungal spores may also vary between species, suggesting that melanized spores (e.g., *Chaetomium* and *Cladosporium*) survive better than hyaline spores [32,33,34]. Furthermore, not all viable spores will germinate and grow on the chosen media; in this case, DG18 and V8. Culture-dependent methods therefore exclude most basidiomycetes (e.g., *Itersonilia*), lichen (e.g., *Scoliciosporum*), and plant pathogenic ascomycetes (e.g., *Taphrina* and *Blumeria*). However, most ascomycetous fungi related to indoor environments (e.g., food products and building materials) are able to grow on DG18 and/or V8 agars [6], which makes the method useful when read by skilled mycologists. 

Another reason for the skewed result may be in the sampling method itself, where the swab for ITS sequencing was taken first followed by the swab for cultivation. The swab for sequencing may contain more viable spores than the swab for cultivation. Secondly, not all viable spores may have been transferred from the swab to the media by simple streaking. An alternative strategy for future studies would be to take the swabs in reverse order and place the cultivation swab in a spore suspension, treat the suspension in ultrasound and spread it onto the media. Another option could be to divide the spore suspension into equal parts and culture one part and sequence the other. A third reason for the difference between culture-independent and -dependent methods is that spore deposits in surface dust may be unevenly distributed across the sample area, especially when there are a low number of reads or CFU. This might be the case with *Asp. niger* in H-3, where the CFU count was larger than the reads. 

Differences in fungal species composition were also seen between dust samples and areas with fungal growth, regardless of detection and identification methods. Some fungal spores are not readily airborne or the source of fungal growth may be too remote from the sampling point. These results show that just because a fungus is not detected, it does not mean that it is not there and again shows that a thorough inspection is particularly important.

### 4.2. Fungal Identification

The paradigm or basic assumption that only 1% of microbes are culturable [35], and therefore identifiable, seems not to apply to the fungal kingdom. All of the OTUs with reads higher than 250 and the majority of OTUs with low reads could be assigned at least a genus identity, suggesting that most fungi found in indoor environments are culturable since they have been named and described morphologically on agar media [6]. Identification to species level was, however, only possible when each sequence was aligned/blasted manually using a combination of curated (UNITE and FunCBS) and un-curated (GenBank) databases. Automated identification of fungi using sequences is only as good as the repository that is used and each of the abovementioned repositories have their strong points. UNITE is good for basidiomycetes, whereas FunCBS is good for food and indoor ascomycetes. GenBank is good for rare and obscure fungi, which are not present in UNITE or FunCBS, but comprises up to 20% misidentifications [36]. Correct naming is also hampered by the adoption of the Amsterdam Declaration [37,38], which has divided the scientific community regarding which names to use and by the discovery of many new species due to genetics and sequence-based phylogeny (e.g., Houbraken et al. [39]). Even though databases such as MycoBank and Index Fungorum are good sources for synonyms and name changes and are up to date with many of the most common genera, correct fungal names are sometimes omitted in the literature (e.g., Reboux et al. [40]) on purpose. Repositories such as GenBank also contain many new entries derived from metagenomics studies with either no fungal identity or uncertain identity of the uncultured fungus. Completely automated assignment of identity to the species level is therefore not possible for a growing number of OTUs, because none of the databases/repositories are fully updated when it comes to the new species, synonyms, and correct names according to the Amsterdam Declaration, which makes laborious manual checking unavoidable. In this pilot study, 117 (9%) out of 1312 OTUs remain uncertain or unidentifiable.

### 4.3. Baseline Spora in a Danish Setting

In order to determine the cause(s) of an unsatisfactory indoor environment, there is a need to know which fungal species are normally present in a home with a satisfactory, problem-free indoor environment. The baseline spora is here defined as the collection of fungal species, which spores are found indoors in dust samples, but not found naturally growing on humid or wet building materials, fabrics, furniture or other materials normally associated with the interior of the building. In other words, the baseline spora is the total spora minus spores from the building mycobiota (building funga). 

Identification to the species level is here essential to distinguish between fungal spores originating from the outside air, dirt, and soil, or food products (the baseline spora of the home) and fungal spores originating from growth on humid or wet building materials inside the building (the mycobiota of the building), particularly species within the genera of *Aspergillus* and *Penicillium* that can be soil- and food-borne as well as building-related [6]. Misidentifying or lumping these species together, combined with inadequate inspection and a lack of home history, may result in the wrong action being taken: health-damaging dampness and fungal growth may be overlooked and the problems may persist or worsen. Alternatively, hygiene or environmental problems may be misconstrued and end in unnecessary and expensive remediation. 

Table 6 gives a conservative bid on a list of fungal species that should be considered the baseline in a Danish setting. Fungal species typically associated with common Danish food products, such as *Asp*. *chevalieri*, *Pen*. *commume*, and *Pen*. *expansum* [6], were not detected in this pilot study, but may also be a part of the baseline spora. Furthermore, species in the *Cla*. *cladosporioides* complex may also belong to the baseline spora, since they are rarely isolated from moldy building materials [28]. A larger, ongoing study will test these hypotheses.

Moreover, the baseline spora may be different from country to country, depending on climate (e.g., cold winters, heavy rainfall or high winds), culture (e.g., type and amount of raw food products, number of potted plants or storage of firewood indoors) and nature (e.g., type and proximity to farming, forests or compost heaps outside). Therefore, other countries’ data and tools, such as the Environmental Relative Moldiness Index (ERMI), which has been developed for dust samples in USA [41], may not work as well in Scandinavia [42]. Baseline fungi must be determined locally and not globally.

## 5. Conclusions

This pilot study presented here suggests that the fungal diversities in Danish homes, which are perceived to have a good and dry indoor environment, have fungal species in common, which could constitute a general Danish baseline spora. A larger study is in progress in order to verify and extend the species list in Table 6. Dust samples may be a good proxy method to examine the indoor air quality of a home and culture-independent methods would give correct fungal identity as well as semi-quantitative amounts. However, two or more sampling areas are necessary to represent larger or multi-story homes together with a questionnaire and two inspections, one during sampling and one after sequencing and data treatment, to fully compare the fungal results with the conditions of the home.

In quality assessment of indoor environments per se, all fungal spores—dead or alive and from outdoors as well as food and building—are important in relation to the health and wellbeing of the occupants. Allergens, glucans, and mycotoxins can be present on all fungal spores and may have health implications if spores are present in high numbers [43]. Daily airing of the home and weekly vacuum cleaning and wiping of all surfaces are important measures that can reduce spore load indoors [44]. Furthermore, if the household comprises asthmatics or allergy sufferers, the number of fungal spores in the home can be reduced even further by having a HEPA filter [45,46] fitted in the vacuum cleaner. 

## Figures and Tables

**Table 1:** Location (zip code and town) and description of the nine private Danish homes (H) sampled from January to March 2015.

Home	Location	Type of Residence	Previous Water Damage	Fungal Growth Detected in 2015
H-1	3460 Birkerød	Flat, mezzanine	None	None
H-2	2800 Kgs. Lyngby	2-storey house	In basement, 2011	None
H-3	2840 Holte	Terraced house	None	Inner wall in bathroom
H-4	3520 Farum	Bungalow	None	Outer wall in bedroom
H-5	2860 Søborg	2-storey house	None	None—dampness in basement
H-6	3200 Helsinge	Bungalow	None	None
H-7	2200 København	Flat, fifth floor	In attic, 2013	None
H-8	2500 Valby	3-storey house	In attic, 2014	None
H-9	2500 Valby	Flat, third floor	None	None

**Table 4:** Culture-independent identification. The most abundant fungal species growing on humid gypsum compared to humid brick wall and the total reads for the different OTUs out and in. High occurrences on building materials compared to total OTUs indoors and outdoors in bold.

Species *	Total DNA Reads		Gypsum Wallboard (GW)				Painted Brick Wall ^§^		
	Out	In	Ua ^†^	Ub ^†^	Pa ^‡^	Pb ^‡^	H-3-G	H-4-Ga	H-4-Gb
*Acr. charticola*	0	0	0	0	856	0	0	**3483**	50
*Aka. lecanii*	0	0	0	0	0	0	0	0	**1289**
*Alt.* sect. *Ulocladium*	0	59	0	0	**2077**	0	0	0	0
*Asp. canadensis*	64	361	0	0	0	0	1	**1856**	0
*Asp. hiratsukae*	1	2	**8123**	**8824**	0	0	1	0	0
*Asp. penicillioides* clade	0	20	0	0	0	98	0	0	0
*Asp.* sect. V*ersicolores*	0	428	0	0	334	80	0	**1620**	**4108**
*Asp. vitricola*	4	813	0	0	0	688	0	0	1
*Can. parapsilosis*	1	1	1	0	0	**16,045**	1	0	0
*Cha. globosum*	19	69	0	235	0	0	0	0	0
*Cla. allicinum*	2700	6909	0	0	0	77	110	0	0
*Cla. dominicanum*	264	140	0	0	6	101	0	476	2
*Cla. halotolerans*	805	72	0	0	124	317	33	14	0
*Cla. sphaerospermum*	29	185	0	0	**4447**	828	0	**8172**	5
*Deb. hansenii*	203	704	0	0	0	0	**94**	**1386**	**1536**
*Exo. lecanii-corni*	0	0	0	0	0	162	0	0	0
*Gib. nigrescens*	10	0	0	0	**5105**	0	0	0	0
*Mey. guilliermondii*	0	221	0	0	0	359	0	0	0
*Mon. tenue*	12	32	0	0	0	0	0	0	**8540**
*Mor. alpina*	214	0	0	0	0	129	0	0	0
*Pen. chrysogenum*	44	909	**8718**	0	41	0	0	1	1
*Pen. corylophilum*	149	0	0	0	0	0	0	0	115
*Pen. roseopurpureum*	0	0	0	0	0	0	0	0	**2156**
*Sta. chartarum*	48	38	0	**4655**	**4477**	0	0	0	1
*Tau. pullulans*	294	87	0	0	0	108	0	0	0
*Wal. muriae*	1	0	0	0	0	90	0	0	0

* Acr.: Acremonium; Aka.: Akanthomyces; Alt.: Alternaria; Asp.: Aspergillus; Can.: Candida; Cha.: Chaetomium; Cla.: Cladosporium; Deb.: Debaryomyces; Exo.: Exophiala; Gib.: Gibellulopsis; Mey.: Meyerozyma; Mon.: Monocillium; Mor.: Mortierella; Pen.: Penicillium; Sta.: Stachybotrys; Tau.: Tausonia; Wal.: Wallemia. ^†^ Untreated gypsum wallboard samples from an experiment on fungal growth in new wallboard conducted at DTU, Kgs. Lyngby, spring 2015 (Andersen et al. [2]). ^‡^ Painted gypsum wallboard samples from a water damaged bathroom in a student accommodation next to DTU Campus, Kgs. Lyngby. Samples were taken the day after sampling at H-2 in Kgs. Lyngby. ^§^ No wallpaper on the brick walls. Samples from Table 3.

**Table 5:** Culture-dependent identification (DG18 and V8). Fungal species detected outdoors and indoors at the nine homes (H). Colony forming units (CFUs) counted and pooled from two DG18 and two V8 plates.

Species *	Total CFUs		Outdoors									Indoors								
	Out	In	H-1	H-2	H-3	H-4	H-5	H-6	H-7	H-8	H-9	H-1	H-2	H-3	H-4	H-5	H-6	H-7	H-8	H-9
*Alt. arborescens*	3	2	0	0	0	1	0	0	0	0	2	0	0	0	0	0	0	0	2	0
*Alt. infectoria*	4	0	0	0	1	0	0	0	0	0	3	0	0	0	0	0	0	0	0	0
*Arthrinium* spp.	0	2	0	0	0	0	0	0	0	0	0	0	0	0	1	0	0	0	1	0
*Asp. fumigatus*	0	2	0	0	0	0	0	0	0	0	0	1	0	0	0	0	0	0	0	1
*Asp. glaucus*	0	3	0	0	0	0	0	0	0	0	0	0	3	0	0	0	0	0	0	0
*Asp. niger*	0	20	0	0	0	0	0	0	0	0	0	0	0	20	0	0	0	0	0	0
*Asp. sydowii*	1	1	0	0	1	0	0	0	0	0	0	0	0	0	0	1	0	0	0	0
*Asp. versicolor*	0	4	0	0	0	0	0	0	0	0	0	0	0	3	0	0	0	1	0	0
*Aureobasidium* spp.	2	0	0	0	0	1	0	0	0	0	1	0	0	0	0	0	0	0	0	0
*Botrytis* spp.	3	0	1	0	2	0	0	0	0	0	0	0	0	0	0	0	0	0	0	0
*Cha. globosum*	2	2	0	0	0	1	1	0	0	0	0	0	1	0	1	0	0	0	0	0
*Cla. allicinum*	28	0	1	0	0	4	0	0	0	0	23	0	0	0	0	0	0	0	0	0
*Cla. cladosporioides*	23	4	1	1	9	0	1	6	0	0	5	0	0	0	0	1	0	1	2	0
*Cla. sphaerospermum*	36	3	0	2	6	13	0	0	1	2	12	0	0	0	0	0	2	0	1	0
*Didymella* spp.	20	0	0	2	0	0	0	0	1	17	0	0	0	0	0	0	0	0	0	0
*Epi. nigrum*	5	0	0	0	1	0	0	0	0	0	4	0	0	0	0	0	0	0	0	0
*Fusarium* sp.	1	0	0	0	0	0	0	0	0	0	1	0	0	0	0	0	0	0	0	0
*Pen. allii*	0	13	0	0	0	0	0	0	0	0	0	0	0	0	3	10	0	0	0	0
*Pen. atramentosum*	3	0	0	0	0	0	0	0	3	0	0	0	0	0	0	0	0	0	0	0
*Pen. brevicompactum*	13	16	1	0	1	0	0	10	0	0	1	2	0	2	1	6	0	0	5	0
*Pen. chrysogenum*	0	5	0	0	0	0	0	0	0	0	0	1	0	0	0	0	0	3	1	0
*Pen. citreonigrum*	0	3	0	0	0	0	0	0	0	0	0	0	0	2	0	0	0	1	0	0
*Pen. crustosum*	4	0	0	0	0	0	0	4	0	0	0	0	0	0	0	0	0	0	0	0
*Pen. decumbens*	1	0	0	0	0	0	0	0	0	1	0	0	0	0	0	0	0	0	0	0
*Pen. digitatum*	2	69	0	0	1	0	0	0	0	1	0	9	0	29	0	31	0	0	0	0
*Pen. echinulatum*	0	1	0	0	0	0	0	0	0	0	0	0	0	0	1	0	0	0	0	0
*Pen. glabrum*	0	15	0	0	0	0	0	0	0	0	0	0	0	1	2	10	0	1	0	1
*Pen. olsonii*	0	7	0	0	0	0	0	0	0	0	0	1	0	0	0	0	0	6	0	0
*Pen. phoeniceum*	2	0	0	0	0	0	0	0	0	0	2	0	0	0	0	0	0	0	0	0
*Pen. polonicum*	0	2	0	0	0	0	0	0	0	0	0	2	0	0	0	0	0	0	0	0
*Pen. spatulatum*	0	1	0	0	0	0	0	0	0	0	0	0	0	0	0	0	0	0	1	0
*Scopulariopsis* sp.	0	1	0	0	0	0	0	0	0	0	0	0	0	1	0	0	0	0	0	0
*Sta. chartarum*	0	1	0	0	0	0	0	0	0	0	0	1	0	0	0	0	0	0	0	0
*Trichoderma* sp.	0	1	0	0	0	0	0	0	0	0	0	0	0	0	0	0	0	0	1	0
Mycelia sterilia	45	25	0	2	5	4	1	2	1	26	4	10	0	4	0	2	0	1	4	4
Yeast	64	2	5	0	11	3	12	1	2	16	14	0	0	0	0	0	0	1	1	0
Total CFUs	262	205	9	7	38	27	15	23	8	63	72	27	4	62	9	61	2	15	19	6

* *Alt.: Alternaria; Asp.: Aspergillus; Cha.: Chaetomium; Cla.: Cladosporium; Epi.: Epicoccum; Pen.: Penicillium; Sta.: Stachybotrys*.

**Table 6:** Baseline fungi (spora) and moisture indicator fungi (mycobiota/funga) in a Danish setting. Current and previous species names, the detection method, and the most common origin are based on this study and studies by Andersen et al. [1] and Samson et al. [6].

Previous Name	Current Name(s)	Detection Method	Substratum/Origin
Baseline fungi			
*Alternaria infectoria*	*Alt. infectoria*	V8/ITS	Cereal plants, grasses/outside
*Aspergillus fumigatus*	*Asp. fumigatus*	DG18/ITS	Wood chip, compost/outside
*Botrytis cinerea*	*Bot. cinerea*	V8/ITS	Onions, cabbage, soft fruit
*Eurotium herbariorum*	*Asp. glaucus*	DG18/ITS	Dried foods, herbs and spices
*Epicoccum nigrum*	*Epi. nigrum*	V8/ITS	Soil, dead plant debris/outside
*Penicillium digitatum*	*Pen. digitatum*	DG18/ITS	Lemons, oranges
*Pen. glabrum*	*Pen. glabrum*	DG18/ITS	Onions
*Pen. roqueforti*	*Pen. roqueforti*	DG18/ITS	Rye bread, blue cheese
Moisture indicator fungi			
*Asp. versicolor*	*Asp. creber,* *Asp. jensenii,* *Asp. versicolor*	V8, DG18/ITS	Most building materials
*Asp. penicillioides*	*Asp. penicillioides,* *Asp. tardicrescens,* *Asp. vitricola*	DG18/ITS	Textiles, leather, paintings, dried grain
*Chaetomium globosum*	*Cha. globosum*	V8/ITS	Gypsum wallboard, plywood
*Cha. murorum*	*Botryotrichum murorum*	V8/ITS	Ceiling tile
*Cladosporium bruhnei*	*Cla. allicinum*	V8, DG18/ITS	Woodwork, plaster, grain
*Cla. sphaerospermum*	*Cla. dominicanum,* *Cla. halotolerans,* *Cla. sphaerospermum*	V8, DG18/ITS	Woodwork, plaster, paint, plywood, textiles
*Pen. chrysogenum*	*Pen. chrysogenum,* *Pen. rubens*	DG18/ITS	Most building materials
*Stachybotrys chartarum*	*Sta. chartarum*	V8/ITS	Gypsum wallboard, cardboard
*Ulocladium alternariae*	*Alt. alternariae*	V8/ITS	Wallpaper, textiles, plant debris
*Wallemia sebi*	*Wal. ichthyophaga,* *Wal. muriae, Wal. sebi*	DG18/ITS	Plaster, brickwork, hay, dried food

## Data Availability

Data are contained within the article or supplementary material. The data presented in this study are available in [Supplementary Table S1].

## Supplementary Materials

The following are available online at https://www.mdpi.com/2309-608X/7/2/71/s1, Table S1: GenBank data from BLAST on selected OUTs.
